# Infection therapy: the problem of drug resistance – and possible solutions

**DOI:** 10.1111/1751-7915.12777

**Published:** 2017-07-24

**Authors:** Harald Brüssow

**Affiliations:** ^1^ Department of Gut Ecology Host‐Microbe Interaction Group Nestlé Research Center Lausanne Switzerland

## Abstract

The rising antibiotic resistance in major bacterial pathogens together with the breakdown of the antibiotic discovery platform creates a critical situation for infection therapy. Recent developments reviving new antibiotic discovery from defining chemical rules for membrane‐passing compounds to isolation chips for soil bacteria and exploring the human microbiome for antibiotic‐producing bacteria are discussed. The potential of bacteriocins, tailocins, phage lysins, phages, probiotics and commensal blends as alternatives to antibiotics is evaluated.

Antibiotic resistance has microbiological, evolutionary, ecological and economical aspects. Its extent can be read from regularly updated reports published by the US and European Centers for Disease Control (https://www.cdc.gov/drugresistance/index.html; http://ecdc.europa.eu/en/eaad/Documents/antibiotics-EARS-Net-summary-2016.pdf). These data show methicillin‐resistant *Staphylococcus aureus* (MRSA) in about 20% of all *S. aureus* isolates from skin and soft tissue infections in Europe, but a stunning 47 per cent for the United States (Rossolini and Mantengoli, 2008; Otter and French, 2010). The CDC and ECDC reports demonstrate similar or higher rates of antibiotic resistance for *Escherichia coli*,* Klebsiella pneumoniae*,* Acinetobacter baumannii*,* Enterococcus faecium*, with rising trends. Today, no antibiotic treatment is possible for a sizable number of patients infected with these multidrug‐resistant ESKAPE organisms (for *E* *nterococcus,* *S* *taphylococcus,* *K* *lebsiella,* *A* *cinetobacter,* *P* *seudomonas,* *E* *nterobacter*; Bassetti and Righi, 2015).

## Breakdown of the antibiotic discovery platform

Then there is an industrial problem with the development of new antibiotics. One aspect is economical: the potentially rapid resistance development against newly marketed antibiotics and their careful, that is limited, use by physicians causes problems with the return of investment for a costly drug development programme. Another aspect is technological, namely the breakdown of the once successful antibiotic discovery ‘Waksman platform’ (Lewis, 2012, 2013, 2017). By overmining soil‐derived actinomycetes, mostly *Streptomycetes*, for antimicrobial activity, the pipeline run dry and was replaced in the 1960s by a strategy modifying existing compounds into active analogues. The following ‘Genomics‐Combichem‐High Throughput‐Rational Drug Design’ phase was a disappointment as it did not yield a single new antibiotic drug even after screening 500 000 synthetic compounds (Payne *et al*., 2007). Antibiotic experts have diagnosed a number of problems. There are only rare chemical compounds that penetrate the lipopolysaccharide‐coated outer membrane of Gram‐negative bacteria, which limits approaches based on the screening of chemical libraries. *In vitro* antibacterial tests are another bottleneck as too many pharmacologically important drug characteristics are not measured in this system. Lewis (2017) mentioned other limitations: for practical and commercial reasons, broad‐spectrum antibiotics were targeted, while species‐selective compounds should be preferred as they avoid collateral damage on the commensal microbiota and cause less selection for widespread resistance development.

## Glimmers of hope

Possible ways out of this dilemma include combination therapies, chemical reworking of antibiotics that were discarded for toxic side‐effects, or targeting highly connected webs of protein and gene interactions in the bacterial cell that go beyond the few classical antibiotic targets, i.e. ribosomes, penicillin‐binding proteins and DNA gyrase/topoisomerase (Brown and Wright, 2016). Furthermore, ninety per cent of natural product chemistry is encoded by silent bacterial operons, whose expression could enable novel discovery platforms (Bentley *et al*., 2002; Laureti *et al*., 2011; Rutledge and Challis, 2015). The Gram‐negative outer membrane barrier might be crossed by permeable prodrugs that are activated inside the bacterial cell by bacterial enzymes. In addition, chemists have recently defined a set of chemical characteristics needed for the penetration of the outer membrane: the compounds must contain an amine, be amphiphilic and rigid. With these rules, they succeeded to convert an antibiotic active against Gram‐positive bacteria for use against *E. coli* (Richter *et al*., 2017). *Caenorhabditis* worms that can be infected with human pathogens by ingestion have been suggested as alternative screening system to *in vitro* tests which offers several *in vivo* characteristics, but can still be adapted to microtitre test format (Moy *et al*., 2006).

## From soil…

Novel antibiotics can also be identified by culture‐independent methods as demonstrated by cloning soil microbial DNA libraries (metagenomes) in *E. coli* (Gillespie *et al*., 2002). Some of the ‘uncultivatable’ soil bacteria can be grown inside diffusion chambers incubated *in situ*, where diffusion provides bacteria with their naturally occurring growth factors (Kaeberlein *et al*., 2002). Subsequently, these devices were developed into isolation chips allowing high‐throughput parallel cultivation platforms (Nichols *et al*., 2010). When such iChip test systems were inoculated with diluted soil samples and placed back into soil and screened after growth for antimicrobial activity on plates overlaid with *S. aureus*, a potent new antibiotic was identified: teixobactin. It bound to lipid precursors of cell wall components, causing lysis of many pathogens from Firmicutes and Actinobacteria. Notably, no resistance evolved against this compound possibly because it targets lipids and not proteins and because the producer strain protects itself against the toxic antibiotic by the physical barrier of the outer membrane and not by a genetically encoded antitoxin system (Ling *et al*., 2015).

## …to the human microbiome

Interbacterial competition is also a characteristic of the human microbiota, making it a possible source for new antibiotics. Indeed, *Staphylococcus lugdunensis*, a competitor to *S. aureus* in the anterior nares of humans, produces a non‐ribosomal peptide lugdunin. It inhibits MRSA and vancomycin‐resistant *Enterococcus* isolates with minimal inhibitory concentrations (MIC) in the micromolar range. Lugdunin inhibits DNA, RNA, protein and cell wall synthesis of *S. aureus*, killing the pathogen without allowing resistance development. Lugdunin is active in an animal infection model (Zipperer *et al*., 2016).

A systematic analysis of > 2000 reference genomes of the human microbiota identified > 3000 small‐molecule biosynthetic gene clusters. The dominant class of small molecules was saccharides, followed by polyketides, non‐ribosomal peptides and modified peptides (Donia *et al*., 2014). To demonstrate the use of this approach, the researchers isolated a thiopeptide from a vaginal *Lactobacillus gasseri* isolate, which inhibited *S. aureus*, pathogenic oral streptococci and vaginal pathogens, but notably not vaginal *Lactobacillus* commensals (Donia *et al*., 2014).

## Alternatives from bacteriocins…

Microbiologists knew for many years bacteriocins, bacterially produced peptides that are active against other bacteria and against which the producer has specific immunity mechanisms. In the past, bacteriocins were technologically explored for food protection, and nisin has been approved as a food preservative. Subsequently, bacteriocins could facilitate the introduction of probiotic bacteria into an already occupied niche (Dobson *et al*., 2012). This concept can be extended against antibiotic‐resistant gut pathogens: *Enterococcus faecalis* replaces indigenous, vancomycin‐resistant enterococci in mice without perturbing commensals (Kommineni *et al*., 2015). However, *in vitro* killing efficacy has not always translated into *in vivo* protection (Dobson *et al*., 2012). *In vivo* efficacy data are still scarce: *Lactobacillus salivarius* protected mice against invasive *Listeria monocytogenes* infection (Corr *et al*., 2007). Thuricin produced by *Bacillus thuringiensis* kills a wide range of *Clostridium difficile* isolates in a colon model (Rea *et al*., 2010) without significant impact on the human gut microbiota (Rea *et al*., 2011). Bacteriocins are also produced by *E. coli* (colicins) or *Pseudomonas* (pyocins), and prevent *E. coli* diarrhoea in pigs and *Pseudomonas aeruginosa* infection in wax moths respectively (Behrens *et al*., 2017). Microcins produced by the probiotic *E. coli* Nissle strain limited the expansion of competing *Enterobacteriaceae* in mice suffering from intestinal inflammation and might thus represent a possible treatment mode for enterobacterial colitis (Sassone‐Corsi *et al*., 2016).

## …to tailocins

Another group of potentially interesting antimicrobials are tail structures from defective phages that function as bacteriocins (tailocins). R‐type pyocins resemble the contractile tail of P2‐like myoviruses, while F‐type pyocins look like the flexible tails from phage lambda. Host specificity is mediated by tail fibres, and when tailocins undergo conformational changes after adsorption to the target bacterium, lethal membrane damage ensues via dissipation of the membrane potential. Efficacy was reported against *P. aeruginosa* in a mouse peritonitis model (Scholl and Martin, 2008). When the tail spike protein of this pyocin was replaced by a coliphage protein (Scholl *et al*., 2009), the engineered pyocin showed protection against *E. coli* infection in a rabbit model (Ritchie *et al*., 2011). Subsequently, pyocins have been engineered that prevented colonization of mice with *C. difficile* (Gebhart *et al*., 2015).

## From phage lysins…

Interesting antimicrobial properties are also displayed by phage lysins (PL; Pastagia *et al*., 2013). PL attack the bacterial cell wall from the inside to release progeny phage. PL lyse Gram‐positive cells also from the outside, while Gram‐negative bacteria are protected by the outer membrane. The modular structure of PL with a catalytic and a binding domain connected by a short linker offers mix and match possibilities for bioengineering to increase solubility, thermostability, binding specificity and catalytic efficiency. Most PL show genus‐restricted killing activity, sometimes species‐restricted killing activity. As they target essential cell wall structures, no PL‐resistant have yet been described. Only non‐neutralizing antibodies develop after *in vivo* application. PL showed a very rapid and efficient *in vitro* lytic activity. *In vivo* activity was described in animal models of pneumonia, endocarditis, pharyngitis, meningitis and sepsis (Pastagia *et al*., 2013). PL eliminated bacterial pathogens from mucosal and skin surfaces and were active against biofilms. Toxic effects are limited to systemic cytokine induction due to release of bacterial debris. Synergistic activity with antibiotics was observed, and PL could even re‐sensitize pathogens to formerly inefficient antibiotics (Daniel *et al*., 2010). Research is underway to extend their use also against Gram‐negative bacteria (Lukacik *et al*., 2012). Overall, this sounds too good to be true. What are potential drawbacks? PL have a relatively high minimal inhibitory concentration (40 μg ml^−1^) (Daniel *et al*., 2010) and a very high‐affinity constant K_a_ = 10^8^ for the cell wall (Loessner *et al*., 2002). This means that after binding and enzymatic action, PL will not diffuse away to renewed action. A solution might be truncated PL consisting only of the catalytic domain. Also the PL binding domain alone can protect mice from MRSA infection (Raz *et al*., 2017). Another problem might be that PL are quickly cleared from systemic circulation (half‐life in rats: 20 min; Entenza *et al*., 2005), which would limit its *in vivo* application, but might be partially compensated by its rapid mode of action.

One might ask why PL in view of these promising activities were not yet tested in clinical trials and whether this represents not a silent argument against their practical value. However, this lack or as in the case of phage therapy, the scarcity of clinical evidence might tell us more about the difficulties to finance product developments with these phage‐based approaches than about objective technical hurdles. In view of the public health importance of these potential alternatives in an era of antibiotic resistance crisis, one might ask why phage and PL trials are not organized by governmental agencies.

## …to phage therapy…

Phages are bacterial viruses, which infect bacteria, produce progeny phage and lyse their target cells. In theory, we have here a self‐amplifying, generally species‐specific antibacterial agent *in situ* produced in proportion to the pathogen level. These are unique pharmacokinetic properties coming close to Paul Ehrlich's magic bullet. Phage therapy has been developed in the Soviet Union as an alternative to antibiotics (Sulakvelidze *et al*., 2001). Today, a multitude of phage products with a wide range of applications are sold as registered, over‐the‐counter products in pharmacies of many countries from the former Soviet Union. Scientific reports on the clinical efficacy of phage therapy go back to the pioneers of phage therapy (d'Herelle with cholera; Brüssow, 2017) or to a large controlled clinical trial of the Eliava Institute in Georgia in the 1960s, which reported a successful prophylaxis trial against *Shigella* dysentery and *E. coli* diarrhoea (Sulakvelidze *et al*., 2001). Beyond these reports, successful published experience with phage therapy is so far limited to small controlled clinical trials (*P. aeruginosa* otitis externa infection) or uncontrolled series of phage applications in Polish patients not responding to antibiotics (Vandenheuvel *et al*., 2015). A controlled clinical trial of *E. coli* diarrhoea in children from Bangladesh showed no advantage of two different phage preparations, including a commercial Russian product, over standard therapy. Subsequent microbiota analysis revealed that *E. coli* pathogen titres in the stool of the patients were probably below the replication threshold for the orally applied phages (Sarker *et al*., 2016). While phage therapy remains an attractive concept, its documentation by clinical trials is still insufficient to confirm its value as an alternative to antibiotics. Phage‐bacterium interaction within the human host is largely undefined, and basic questions lack still an answer: What bacterial infections are suitable targets for phage therapy? What phage types present suitable *in vivo* properties for clinical trials?

## …and bacteriovores

The current overview started with small chemical compounds (antibiotics), continued with proteins (bacteriocins, lysins), protein complexes (tailocins) and viruses (bacteriophages) as antimicrobial agents. This gradient of biological complexity for antimirobial agents can be extended to viable bacteria. A particularly fascinating approach is bacteria eating bacteria (bacteriovores). The *Bdellovibrio* and *Micavibrio* bacteriovores represent such living antimicrobial agents. Bacteriovores seem to be safe: Rectal bacteriovore application in rats induced only a modest cytokine response and had minimal effects on faecal microbiota composition (Shatzkes *et al*., 2017b). Zebrafish infected with *Shigella flexneri* and exposed to *Bdellovibrio* showed an initial bacterial predation phase and a subsequent elimination phase of the bacteriovore by host neutrophils (Willis *et al*., 2016). Upon intranasal inoculation in rats, the predator bacteria reduced *K. pneumoniae* burden in the lungs by 1000‐fold (Shatzkes *et al*., 2016). In contrast, predator bacteria were unable to reduce *K. pneumoniae* titres in the blood of intravenously infected rats (Shatzkes *et al*., 2017a). Initial claims for treatment effects against keratitis of the eyes were not confirmed (Boileau *et al*., 2016), and clinical exploration has thus not yet started.

## Outlook

So far, all alternative anti‐infectious agents described in the present overview were selected for their lytic action on pathogenic bacteria. Alternative approaches also need to be considered such as measures reducing the colonization level with antibiotic‐resistant bacteria like MRSA before it comes to an infection. Ecological interventions reducing the transmission of antibiotic‐resistant bacteria belong into this category as well as means to modify the expression of virulence factors by these bacteria. Ecological theory might thus identify novel treatment approaches against polymicrobial diseases with antibiotic‐resistant pathogens (Quinn *et al*., 2016). At the end, I want to mention one such approach: *Lactobacillus paracasei* strain ST11 showed therapeutic effects in a controlled clinical trial against childhood diarrhoea (Sarker *et al*., 2005) in a country where diarrhoea‐associated bacteria are largely antibiotic‐resistant (Jiang *et al*., 2002). Unfortunately, the mode of probiotic action is only poorly defined (Servin, 2004). Also a clinical trial with a defined cocktail of bacterial gut commensals showed efficacy against *C. difficile* infection (Tvede and Rask‐Madsen, 1989). It is thus likely that we will see more such clinical trials with probiotics or commensal cocktails in infection therapy (Liévin‐Le Moal and Servin, 2014). The next level of complexity for antimicrobial agents has already been documented, namely the transfer of an entire microbiota from a healthy subject to *C. difficile* patients (van Nood *et al*., 2013). These approaches open a new vista where pathogens are not targeted for lysis, but are put under competitive ecological pressure (Brüssow, 2007). As these interactions are complex in nature, it can be hoped that they are less prone to subversion by resistance development of pathogens.

## Conflict of interest

None declared.
